# Post-ictal Modulation of Baroreflex Sensitivity in Patients With Intractable Epilepsy

**DOI:** 10.3389/fneur.2018.00793

**Published:** 2018-09-26

**Authors:** Behnaz Esmaeili, Farhad Kaffashi, Wanchat Theeranaew, Aman Dabir, Samden D. Lhatoo, Kenneth A. Loparo

**Affiliations:** ^1^Department of Neurology, Columbia University Medical Center, New York, NY, United States; ^2^Department of Electrical Engineering and Computer Science, Case School of Engineering, Case Western Reserve University, Cleveland, OH, United States; ^3^Epilepsy Center, Neurological Institute, University Hospitals Case Medical Center, Case Western Reserve University, Cleveland, OH, United States

**Keywords:** epilepsy, autonomic nervous system, baroreflex function, baroreflex sensitivity, heart rate variability, SUDEP

## Abstract

**Objective:** Seizure-related autonomic dysregulation occurs in epilepsy patients and may contribute to Sudden Unexpected Death in Epilepsy (SUDEP). We tested how different types of seizures affect baroreflex sensitivity (BRS) and heart rate variability (HRV). We hypothesized that BRS and HRV would be reduced after bilateral convulsive seizures (BCS).

**Methods:** We recorded blood pressure (BP), electrocardiogram (ECG) and oxygen saturation continuously in patients (*n* = 18) with intractable epilepsy undergoing video-EEG monitoring. A total of 23 seizures, either focal seizures (FS, *n* = 14) or BCS (*n* = 9), were analyzed from these patients. We used 5 different HRV measurements in both the time and frequency domains to study HRV in pre- and post-ictal states. We used the average frequency domain gain, computed as the average of the magnitude ratio between the systolic BP (BPsys) and the RR-interval time series, in the low-frequency (LF) band as frequency domain index of BRS in addition to the instantaneous slope between systolic BP and RR-interval satisfying spontaneous BRS criteria as a time domain index of BRS.

**Results:** Overall, the post-ictal modulation of HRV varied across the subjects but not specifically by the type of seizures. Comparing pre- to post-ictal epochs, the LF power of BRS decreased in 8 of 9 seizures for patients with BCS; whereas following 12 of 14 FS, BRS increased. Similarly, spontaneous BRS decreased following 7 of 9 BCS. The presence or absence of oxygen desaturation was not consistent with the changes in BRS following seizures, and the HRV does not appear to be correlated with the BRS changes. These data suggest that a transient decrease in BRS and temporary loss of cardiovascular homeostatic control can follow BCS but is unlikely following FS.

**Significance:** These findings indicate significant post-ictal autonomic dysregulation in patients with epilepsy following BCS. Further, reduced BRS following BCS, if confirmed in future studies on SUDEP cases, may indicate one quantifiable risk marker of SUDEP.

## Introduction

Autonomic phenomena are well described primary manifestations of epileptic seizures (1). Secondary autonomic alterations such as tachycardia are common peri-ictal phenomena (2, 3), although it is a combination of profound autonomic (bradycardia, asystole) and respiratory (bradypnea, apnea) changes that appear to comprise the agonal pathophysiologic mechanisms leading to Sudden Unexpected Death in Epilepsy (SUDEP) (4). The role of brainstem control structures in such dysfunction, driven by seizure related activation of the autonomic nervous system regulating cortical areas such as the insula, anterior cingulate gyrus, and ventromedial prefrontal cortex, is likely to be critical although direct measures of brainstem homeostasis in human seizures are a challenge. The baroreflex, a cardiovascular feedback control mechanism devoted to the maintenance of circulatory homeostasis, reflects autonomic control mediated by medullary autonomic neural circuits (5). Polygraphic studies in the epilepsy monitoring unit (EMU) using several simultaneous physiologic measurements (electroencephalography [EEG], 3-channel electrocardiography [ECG], pulse oximetry, respiration, and continuous noninvasive blood pressure [BP]) provide an opportunity to address this challenge in part through examination of baseline, ictal, and post-ictal baroreflex sensitivity (BRS) calculations.

Several studies have reported impaired BRS in epilepsy patients in the inter-ictal, non-seizure state. Greater BP variability and reactivity in patients with focal epilepsy compared to normal controls (6), suggesting decreased BRS and autonomic variability in patients with temporal lobe epilepsy (7) have been described in the inter-ictal state as well as during provocative maneuvers such as Valsalva and tilting (8). Post-ictal modulation of blood pressure varies after focal seizures suggesting possible autonomic imbalance (9). Although impaired ictal BRS has been noted in rodent seizure models (10), similar ictal and post-ictal studies in humans are unavailable, in large part because reliable, continuous peri-ictal BP recordings are difficult to obtain in clinical practice. This is an important gap in knowledge because deviant BRS responses, particularly those associated with hypotension (11), may help premortem identification of patients most at risk of SUDEP.

Recently, researchers identified the BRS as an indicator of autonomic dysfunction caused by bilateral convulsive seizures (BCS) using the spectral computation method (12). The computation for spectral BRS depends on both BP and heart rate (HR), and without carefully studying the variability in HR any quantifiable variations in BRS could easily be mistaken for variability in spectral HR measures. Heart rate variability (HRV) measures were not analyzed in the previous study, so this work addresses this issue and also includes the analysis of spontaneous BRS to further strengthen the results and conclusions. Specifically, we asked how seizures affect BRS in the post-ictal state. We hypothesized that BRS would be reduced in the post-ictal state. Generalized tonic-clonic seizures are the greatest risk factor for SUDEP; SUDEP most often occurs after this type of seizure. We focused on changes in BRS after BCS compared to focal seizures (FS); we further hypothesized that BRS would be reduced after BCS. We also tested how changes in BRS correlate with post-ictal EEG changes known to correspond with autonomic dysfunction. We further verified our findings by analyzing HRV and showing that the changes in BRS were not the result of variability in HR.

### Subjects

The Institutional Review Board at Case Western Reserve University approved the study, and written informed consent in accordance with the Declaration of Helsinki was obtained from all participants. We studied 9 BCS and 14 focal seizures (FS) including 2 focal seizures with impaired awareness in 18 patients (8 women and 10 men aged 17–67 years, mean 35 ± 3.6). Seventeen patients were right-handed and 1 was left-handed. All patients had intractable epilepsy, with frequent breakthrough seizures; they were admitted for in-patient video-EEG monitoring for better seizure characterization and/or pre-surgical assessment. Neurological and general physical examinations were normal; none had clinical signs of autonomic dysfunction and none were being treated with drugs known to interfere with autonomic function. The etiology of epilepsy was structural with medial temporal lobe/hippocampal sclerosis in one patient while the etiology of epilepsy for the other 17 patients remained unknown. Patients were on various anti-seizure drug combinations, which were similar between the two groups. Anti-seizure medications (including Carbamazepine, Clonazepam, Lacosamide, Lamotrigine, Levetiracetam, Lorazepam, Oxcarbazepine, Phenytoin, Topiramate, Valproic acid, and/or Zonisamide) were tapered or withdrawn in all patients in order to provoke seizures. Bilateral convulsive seizures included: 3 generalized onset tonic-clonic seizures, two unknown onset tonic-clonic seizures and 4 focal to bilateral tonic-clonic seizures. Focal seizures included: focal onset tonic, clonic, myoclonic, or automatism. Seizure onset zone in focal onset seizures included temporal, frontal or parietal lobes.

### Recording

Polygraphy was recorded in the EMU: simultaneous 24 h 3-channel ECG using Neurofax EEG-1100A (Nihon Kohden Tokyo, Japan), oximetry with Oximax N-600X (Covidien PLC, Dublin, Ireland), respiration using Ambu Sleepmate abdominal and thoracic belts (Ambu, Copenhagen, Denmark), and continuous beat-to-beat noninvasive BP recordings with CNAP (CNSystems Medizintechnik AG, Graz, Austria) (11). Data was collected at 200 samples *per second* and band pass filtered before analog to digital conversion to prevent aliasing and to eliminate low frequency trends. The analog low pass filter had a cutoff frequency of 70 Hz and the high pass filter had a time constant of 10 s. Data was then reviewed and analyzed in custom software developed by our group in Matlab (The MathWorks, Inc., Natick, Massachusetts, United States). EEG was recorded from the standard 10–20 system scalp locations, except in seven patients who had both scalp and intracranial electrodes. The EEG data recorded from the intracranial electrodes was not used in this study.

### Signal processing

Video/EEG recordings with BP and ECG measurements were processed to identify epileptic events including the pre-ictal and post-ictal periods that were free of movement artifact. The recorded signals were then visually analyzed and annotated by an expert epileptologist to determine different electroclinical phases including: the onset and offset of the EEG seizure, tonic and clonic phases; post-ictal generalized EEG suppression; post-ictal EEG slowing and EEG return to the baseline state. Annotated signals were then visualized in a custom developed graphical user-interface (GUI) in Matlab that included the signal processing tools that were used for the data analysis. The first step in the data analysis was to automatically detect ECG R-waves and analyze BP waveforms to automatically detect the systolic peak and quantify the systolic BP (BPsys). All automatically detected ECG and BP events were reviewed and visually confirmed.

### Baroreflex function and heart rate variability

Baroreflex sensitivity (BRS) was studied by analyzing the relationship between spontaneous fluctuations of the BPsys and corresponding HR fluctuations (i.e., RR-interval), that reflect input and reflex output activities of the baroreflex, respectively (7). The time series data was converted to the frequency domain using the Lomb–Scargle periodogram (13–15), and the spectral BRS was calculated as the average of the magnitude ratio (transfer function) between oscillations of BPsys and RR-interval in the low frequency (LF) range (i.e., 0.04–0.15 Hz), provided there was sufficient coherence (>0.2), a conventional criterion to guarantee reliable estimates of the magnitude ratio (gain) (16). Spectral BRS was calculated for 2- and 5-min epochs at baseline and immediately after seizures. On the other hand, the sequence method was used to calculate the spontaneous BRS for 30 min of data during baseline and immediately after seizures (17, 18). RR-intervals and BPsys were scanned automatically to identify spontaneous sequences consisting of three or more consecutive beats in which BPsys progressively increased and RR-interval progressively lengthened or BPsys progressively decreased and RR-interval progressively shortened. Then, the regression between the BPsys values and the RR-interval values was calculated for each sequence and the correlation coefficient of the regression fit was estimated. Spontaneous BRS was estimated using the regression slope between the RR-interval and BPsys if the correlation coefficient was >0.8. Otherwise, the sequence was rejected and not included in the statistical analysis.

In the time domain, heart rate variability (HRV) is measured by computing the beat-to-beat (NN) variability (19). We selected three commonly used time domain computations including standard deviation of all NN intervals (SDNN), the square root of the mean of the sum of the squares of differences between adjacent NN intervals (RMSSD), and standard deviation of differences between adjacent NN intervals (SDSD). In the frequency domain, data from the spectrum that has also been used in the BRS calculation can be used to compute another measure for HRV. In addition to LF (0.04–0.15 Hz) power, we also computed the average power in the high frequency range (i.e., 0.15–0.4 Hz) for the HRV computation. We compared the HRV in 5-min epochs of ECG in the pre-ictal baseline state with HRV in 5-min epochs immediately following a seizure.

In 9 BCS, we measured post-ictal generalized EEG suppression (PGES) and we evaluated the correlation between PGES duration and BRS values and the duration of oxygen desaturation below 90%. PGES was defined as the immediate (during 30 s) post-ictal, generalized absence of electroencephalographic activity >10 μV in peak-to-peak amplitude, which allows for muscle, movement, breathing, and electrode artifacts (20).

### Data analysis

Data are presented as mean ± SEM unless otherwise stated. Data were analyzed using one-way ANOVA with repeated measures, with time as the within-subject factor. Multiple comparisons were analyzed using two-way ANOVA, with the epileptic event as the between-subject factor and time as the within-subject factor. Where necessary, *post-hoc* analysis was carried out with Tukey's multiple comparison tests to determine changes in power. Significance was set at *p* < 0.05. Data were analyzed using Matlab and R.

## Results

**Figure 1:**
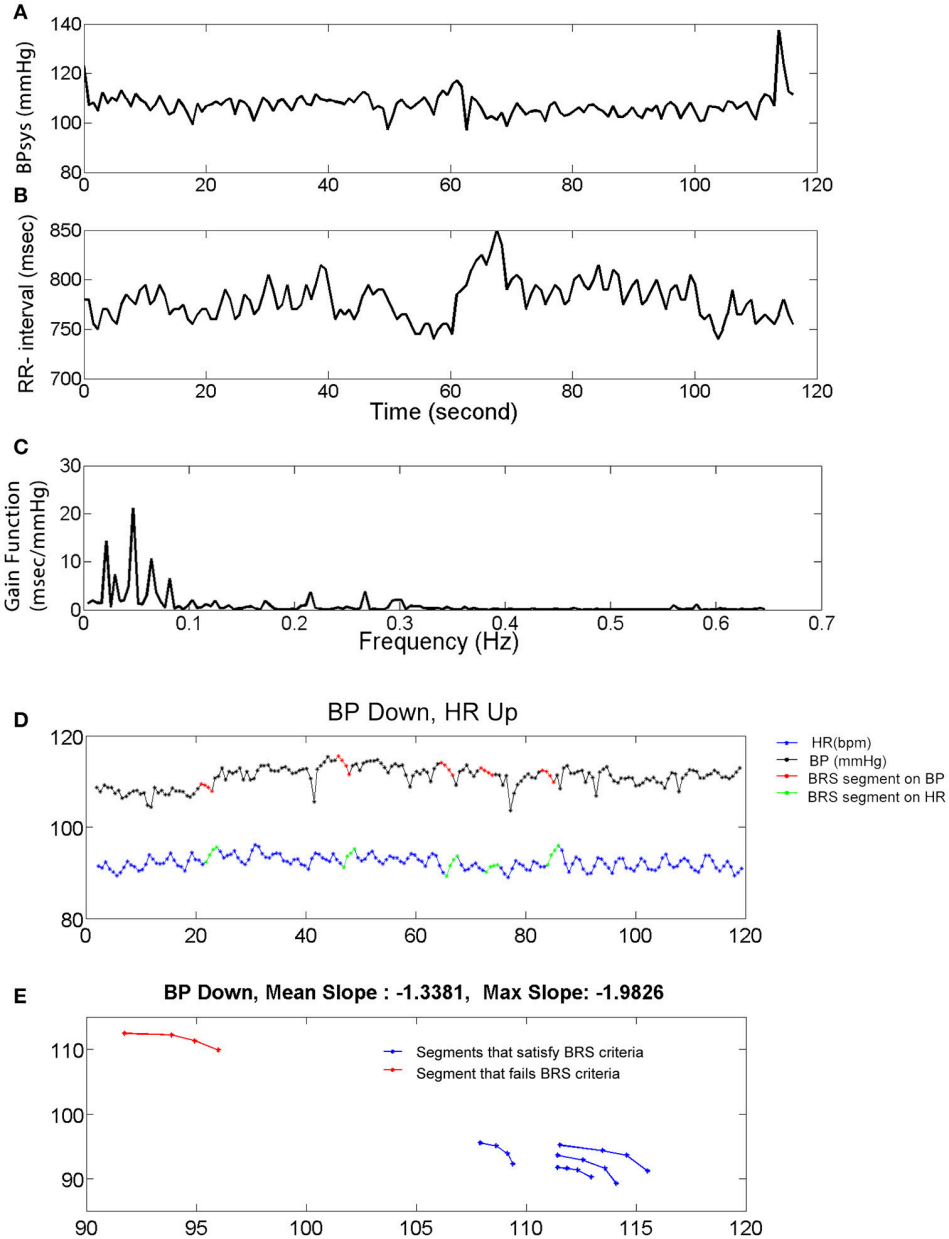
Systolic blood pressure (BPsys), RR-interval, and baroreflex sensitivity (BRS). **(A,B)** A 2 min epoch or time series of BPsys **(A)**, and RR-interval **(B)** in a representative subject. **(C)** If coherence between BPsys and RR-interval was >0.2, then BRS was calculated as the average of the transfer function gain between oscillations of BPsys and RR-interval in the LF range (0.04–0.15 Hz). **(D,E)** The 4 points sequence of decreasing BPsys and corresponding increasing HR response automatically were detected and plotted vs. each other. Then a line was fitted to each detected segment (BPsys vs. HR) and if the goodness of fit, R-Square, was greater than 0.8, the slope was accepted as estimated spontaneous BRS value of that segment.

We evaluated the effect of BCS and FS on autonomic drive measuring HR, BP, HRV, and BRS for 23 epileptic seizures. Results were plotted separately for each seizure as the time series of BP and HR, including the average magnitude ratio of BPsys and RR-interval (spectral BRS), and the spontaneous changes of BRS (Figure 1). Results for HRVs in both the time and frequency domains are shown separately in Tables 1–**3**.

**Table 1:** Effect of bilateral convulsive seizures (BCS) on heart rate variability (HRV).

	**Mean difference**	**SD**	***F*_(1, 17)_**	***p*-value**
**BCS: Post-ictal vs. Baseline**				
SDNN (s)	−0.067	0.305	0.436	0.519
RMSSD (s)	−0.120	0.422	0.666	0.427
SDSD (s)	−0.120	0.423	0.666	0.426
HF (s^2^)	−0.029	1.141	0.004	0.952
LF (s^2^)	−0.534	3.492	0.289	0.598

*Overall, the mean value of HRV decreased after BCS, however, the difference between post-ictal and baseline values was not statistically significant in the current set of data*.

### Systolic blood pressure and heart rate after BCS and FS

**Figure 2:**
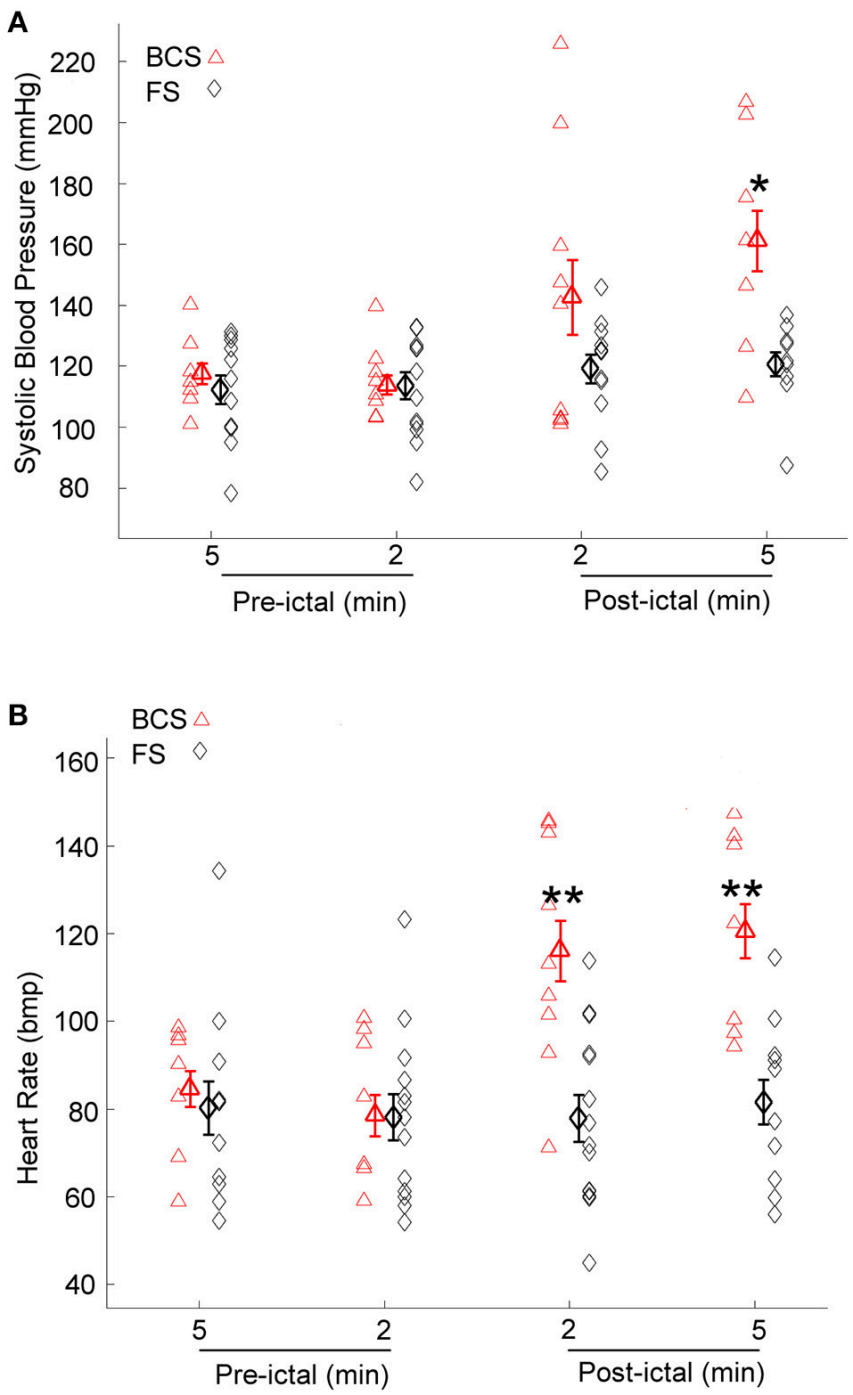
Effect of bilateral convulsive seizures (BCS) and focal seizures (FS) on systolic BP (BPsys) and heart rate (HR). **(A)** Following BCS, the mean value of BPsys significantly increased compared to pre-ictal baseline values and also compared to FS. These findings suggest either activation of the sympathetic drive or suppression of the parasympathetic activity or the compromised autonomic control system that reduces the fluctuations in BP during normal condition. (^*^p < 0.05 compared with that of FS; Two-way ANOVA, Tukey's multiple comparison tests). **(B)** HR values significantly increased following BCS compared to pre-ictal baseline values, whereas after FS there was no significant change in HR (One-way repeated-measures ANOVA). Mean values of HR following BCS were significantly higher than that of FS (^**^p < 0.01 compared with that of FS; Two-way ANOVA, Tukey's multiple comparison tests).

Immediately after BCS and FS, BPsys changed to values higher or lower than at baseline. Mean value of BPsys during 5-min epochs immediately after BCS was significantly higher than mean value of BPsys during the baseline recordings [One-way repeated-measures ANOVA; *F*_(3, 28)_ = 4.03, *p* < 0.05, *n* = 9] (Figure 2A). There was no significant difference between the mean values of BPsys immediately after FS compared to the pre-ictal state [One-way repeated-measures ANOVA; *F*_(3, 41)_ = 0.645, *p* > 0.05] (Figure 2A). The mean value of BPsys was significantly higher following BCS compared with that of FS [*F*_(1, 70)_ = 8.978, *p* < 0.01] (Figure 2A).

HR increased immediately following 8 of the 9 BCS. Heart rate decreased within 2 min after BCS in one patient by 25% compared to baseline values. The mean value of HR increased significantly after BCS in all subjects [One-way repeated-measures ANOVA; *F*_(3, 27)_ = 7.506, *p* < 0.001, *n* = 9] (Figure 2B). HR values were not significantly influenced by FS (One-way repeated-measures ANOVA; *F*_(3, 44)_ = 0.087, *p* > 0.05) (Figure 2B). The mean value of HR was significantly higher after BCS compared with FS [Two-way ANOVA; *F*_(1, 72)_ = 18.818, *p* < 0.001]. *Post-hoc* comparisons showed significantly increased HR in periods of 2 and 5 min periods after BCS compared with FS (*p* < 0.01) (Figure 2B).

### Heart rate variability after BCS and FS

Overall, average values of HRV decreased following BCS compared to the pre-ictal baseline values (Table 1). However, the post-ictal changes in HRV, compared to pre-ictal values, varied across the different HRV measurements and subjects. HRV decreased following approximately half of the BCS while it increased following the rest of them. In FS group, average values of HRV slightly decreased following seizures compared to baseline except for LF power (Table 2). Similar to BCS, post-ictal modulation of HRV varied across subjects and specific HRV measurements. Our results did not show any significant difference between post-ictal HRV values compared to pre-ictal baseline in both BCS and FS groups. (Tables 1, 2). Further, there was no significant difference between changes in HRV following BCS compared to FS (Table 3).

**Table 2:** Effect of focal seizures (FS) on heart rate variability (HRV).

	**Mean difference**	**SD**	***F*_(1, 27)_**	***p*-value**
**FS: Post-ictal vs. Baseline**			
SDNN (s)	−0.004	0.023	0.028	0.869
RMSSD (s)	−0.007	0.025	0.120	0.732
SDSD (s)	−0.007	0.025	0.117	0.735
HF (s^2^)	−0.015	1.168	0.002	0.968
LF (s^2^)	0.310	5.475	0.052	0.822

*The mean values of HRV decreased after FS in majority of HRV measurements, however, the difference between post-ictal and baseline values was not statistically significant*.

**Table 3:** Comparison of the heart rate variability (HRV) after BCS and FS.

	***F*_(1, 22)_**	***p*-value**
**BCS vs. FS**		
SDNN (s)	0.529	0.476
RMSSD (s)	0.869	0.363
SDSD (s)	0.871	0.362
HF (s^2^)	0.001	0.978
LF (s^2^)	0.163	0.691

*The HRV showed a decreasing trend after BCS and FS compared with baseline values, however, the difference between BCS and FS was not statistically significant in the current set of data*.

### Baroreflex sensitivity after BCS and FS

**Figure 3:**
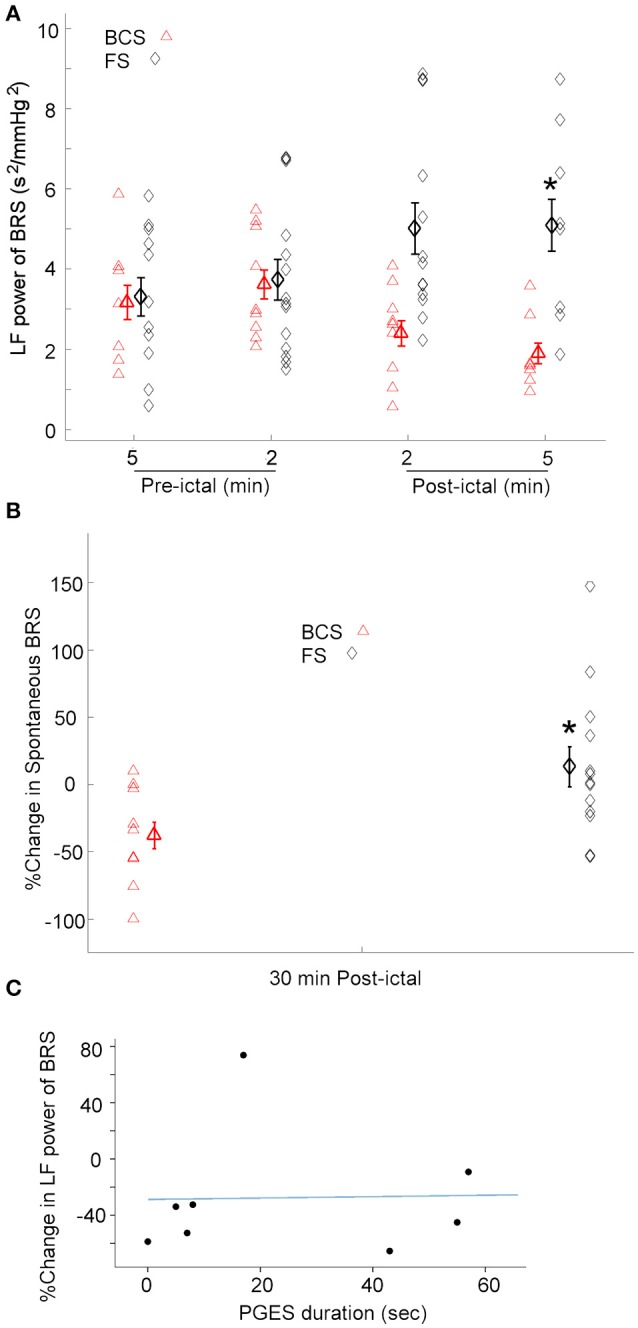
BRS following BCS, FS, and PGES. **(A)** LF power of the BRS decreased after BCS compared to the FS indicating compromised BRS in BCS. This suggests the transient impairment of the central autonomic control of the cardiovascular system immediately after BCS. (^*^p < 0.05 compared to the BCS, Two-way ANOVA; Tukey's *post-hoc* test). **(B)** Similarly, spontaneous BRS decreased after BCS compared to the FS during 30 min post-ictal (^*^p < 0.05 compared to the BCS). **(C)** Scatter plot shows no significant correlation between post-ictal percent change of baroreflex sensitivity and PGES duration.

Given the heterogeneity of autonomic changes during ictal and post-ictal states, data was divided based on the predominant response trends observed. During 8 of 9 BCS, there was a decrease in LF power of BRS in the 5 min immediately after seizures [One-way repeated-measures ANOVA; *F*_(3, 24)_ = 4.569, *p* < 0.05, *n* = 8]. Whereas, in one subject, BRS increased immediately after seizures by 79% compared with baseline values (Figure 3A). Similarly, spontaneous baroreflex sensitivity decreased during the 30 min after the seizure for a majority of BCS (mean percent change −50.3 ± 12.0; *n* = 7). Spontaneous BRS remained unchanged after one BCS and increased by 9% in another patient. Spontaneous BRS during the 30 min after BCS significantly decreased compared to values 30 min before BCS [t_(6)_ = 2.452, *p* < 0.05] (Figure 3B).

The effect of FS on BRS was also tested. In 12 of 14 FS, the low frequency power of BRS in 2 and 5 min epochs after seizures increased significantly compared to the baseline [One-way repeated-measures ANOVA; *F*_(3, 35)_ = 3.207, *p* < 0.05] (Figure 3A). However, in two subjects, BRS decreased immediately after seizure by 78 and 50% compared with baseline values. These results suggest that baroreflex control of the cardiovascular system increased during most FS studied here, indicating enhanced coupling between BPsys and RR-interval and blunted fluctuations in BP. Spontaneous BRS increased during 30 min after 8 FS (mean percent change = 42.9 ± 17.5). Whereas, spontaneous BRS decreased after 5 FS, including two focal seizures with impaired awareness (mean percent change = −32.5 ± 8.7). Our results showed spontaneous BRS significantly increased during 30 min after the majority of FS [t_(7)_ = 2.512, *p* < 0.05, *n* = 8] compared to baseline values.

We compared the effects of BCS on BRS with FS in 2 and 5 min epochs after all seizures (Figure 3A). A two-way ANOVA on BRS values showed a significant difference between BCS and FS [*F*_(1, 71)_ = 11.670, *p* < 0.01] (Figure 3A). BCS had decreased BRS compared with FS; the difference between BCS-induced changes in BRS and that of FS in the 2 min immediately after seizure was statistically significant (*p* < 0.05; Tukey's *post-hoc* test). In two subjects who had both BCS and FS recorded, FS did not show the same trend of changes as BCS in the same patient and in comparison to the BCS of other patients; changes were similar to the FS in other patients. Similarly, spontaneous BRS decreased significantly during the 30 min after BCS compared to that of FS (t(20) = 2.416; *p* < 0.05) (Figure 3B).

### Post-ictal EEG suppression and oxygen desaturation

Because the above findings indicate that BCS cause sympathetic/parasympathetic-driven BRS fluctuations, and an association between post-ictal generalized EEG suppression (PGES) and autonomic changes (21) has been reported, we examined possible correlation between the post-ictal BRS profile in BCS patients and the duration of PGES. PGES durations varied between 0 and 88 (mean = 34.12 ± 10.4) s. In 8 BCS, we assessed the correlation between PGES duration and post-ictal BRS percent change. There was no significant correlation between PGES duration and BRS percent change [*F*_(1, 7)_ = 0.121, *r*^2^ = 0.017, *p* > 0.05; Figure 3C].

Review of oxygen saturation data acquired during continuous video-EEG recordings confirmed that no patient had oxygen desaturation below 90% during and after FS. Oxygen saturation data acquired during continuous monitoring showed oxygen desaturation below 90% in 8, and below 70% in 7 BCS. Mean duration of oxygen desaturation below 90% was 82.6 ± 18.5 s (*n* = 8). Our results did not show any significant correlation between the duration of PGES with duration of oxygen desaturation in the current set of data [*F*_(1, 6)_ = 0.303, *r*^2^ = 0.048, *p* > 0.05].

## Discussion

The strongest risk factor for SUDEP is poorly controlled generalized tonic-clonic seizures and SUDEP usually occurs after generalized convulsive seizures. We used a simple experimental design and polygraphy data obtained from epilepsy patients in the EMU to compare the effect of BCS and FS on BP, HR, and O2 saturation in the post-ictal state. We further investigated how BCS affects BRS and HRV compared to FS in post-ictal state. We found autonomic activity is more profoundly altered after BCS than after FS as evidenced by significant changes in HR and BP values. In BCS, there was a significant increase in mean values of HR and BP compared to baseline values and that of FS. Previous studies have reported ictal and post-ictal tachycardia and decreased post-ictal heart rate variability, in addition to abnormal shortening or prolongation of QT interval in some patients with BCS (22–25). Measuring electrodermal activity, another study has shown prolonged increased sympathetic activation and reduction of cardiac vagal influence following seizures (21). HR and BP are influenced by the balance between sympathetic and parasympathetic drives, although separate quantification of either drive is difficult (26). However, high frequency power of HR denotes parasympathetic modulation of the heart rate, and low frequency power of the BP is representative of sympathetic modulation of BP. Our results, in the current set of data, showed heterogeneity in post-ictal autonomic changes within FS and BCS groups, compared to FS, low frequency power of BP increased after BCS (data not presented). These findings suggest dominant sympathetic- and/or diminished parasympathetic-driven modulation of HR and BP signals following BCS, which causes increased blood pressure and tachycardia. Several studies indicate attenuated parasympathetic activity as a predisposing factor for cardiac arrhythmias and sudden death (27–29), whilst enhanced vagal activity is conversely protective against lethal arrhythmias (30, 31). The cohort presented here does not include any SUDEP cases, indicating that our result cannot imply any direct correlation or causation related to SUDEP. However, the pattern of results presented shows that autonomic nervous system alterations following BCS differ significantly from FS, which helps to clarify at least in part an important observation in SUDEP, that death is vastly more common in BCS patients. An interesting aspect of our findings is that BRS was decreased after the majority of BCS. This suggests important differences in pathophysiological responses between the BCS and FS groups and raises the possibility that only BCS are of sufficient severity to dysregulate autonomic centers, which critically interfere with the baroreflex function, cardiovascular homeostasis and potentially lead to death. By also examining changes in HRV in these patients, we are able to confirm that the measured changes in BRS (spectral and spontaneous) are not due to changes in HRV, but due to impaired BRS, which is described in more detail next.

We found that LF power of the BRS decreased in 7 of 9 patients with BCS during the 5 min in the post-ictal state. However, our results showed an increase in BRS in 12 of 14 FS immediately following the seizure. Decreased BRS after BCS indicates decoupling or decreased coupling between the input and output arms of the baroreflex, and that there is briefly impaired central autonomic regulation after BCS; which may result in increased vulnerability of BCS patients to SUDEP. Furthermore, spontaneous BRS decreased during 30 min after 7 of 9 BCS compared to pre-ictal values, as opposed to increased spontaneous BRS in 8 of 14 FS. Compromised post-ictal BRS is indicative of an inability to maintain BP in a given range, resulting in larger changes in BP and HR, as seen in our patients. It therefore follows that insufficient or delayed compensatory responses to decreased BP can precipitate systemic or cerebral blood supply compromise and consequent tissue hypoxemia. Conversely, effective baroreflex functioning reduces fluctuations in BP. In BCS with compromised BRS, as one would expect, BP fluctuations were greater and lasted longer. We previously reported immediate post-ictal hypotension (11) in BCS; peri-ictal hypotension associated with impaired BRS may contribute to SUDEP; since in such cases impaired baroreflex function would delay compensatory increases in BP and cause cerebral blood flow compromise. The high incidence of compromised BRS and hemodynamic instability in BCS compared to FS, and the relatively low incidence of SUDEP in the epilepsy population suggests that the presence of one autonomic dysregulatory factor may be necessary but by itself insufficient to cause SUDEP.

We confirmed that both BCS and FS affect sympathetic and parasympathetic drives resulting in HRV changes after seizures. Post-ictal changes in HRV following both BCS and FS are not unidirectional. There was no significant difference between post-ictal HRV in BCS and FS groups in the current data, confirming that impaired BRS following BCS is not a direct result of seizure-induced changes in HRV. In addition, another important aspect of our findings is the heterogeneous pattern within the BCS and FS groups; not all BCS cause decreased BRS, even in the same patient; this may reflect the heterogeneity of the mechanisms underlying SUDEP, although we have shown that impaired BRS occurs more frequently in patients with BCS compared to FS, even after non-fatal seizures.

We observed oxygen desaturation below 90% in 8 and below 70% in 7 BCS with mean duration of oxygen desaturation below 90% of 82.6 s. None of the FS had oxygen desaturations below 90%. Increased HR after BCS, consistent with other studies, may contribute to cardiac injury resulting in lethal cardiac arrhythmia (32). The hypoxemia seen in BCS, coupled with increased ictal catecholamine release may affect cardiac re-polarization and contribute to cardiac arrhythmias (33, 34).

Differences in both degree and duration of the described measures of autonomic dysregulation may in part help explain why generalized tonic-clonic seizure is a major risk factor for SUDEP. Previous works have reported that severe PGES is associated with SUDEP (20). poh et al (21) showed strong correlation between PGES and both sympathetic and parasympathetic alterations following generalized tonic-clonic seizure. Here, 7 of 9 BCS were followed by PGES. Our results did not show significant correlation between duration of PGES and BRS values, indicating that the presence of PGES is associated with severity of autonomic changes although not quite in the way expected. Also, our results did not show any significant correlation between duration of PGES and duration of hypoxemia, in the current set of data. Prolonged PGES (>50 s) has been reported in patients with refractory epilepsy, patients at high risk of SUDEP (20). Previous works have consistently found that generalized convulsive seizures with prolonged PGES (i.e., >20 s) have significantly higher sympathetic activation and parasympathetic reduction than seizures with short PGES (21). It is possible that seizures with prolonged PGES may not follow the trend observed here and catastrophic BRS failure may be more likely in patients with prolonged PGES.

The focus of this study was to determine how BCS and FS affect BRS in the post-ictal state. We recognize the limitations in our study. The design may seem underpowered for within-group comparison because there are just 9 and 14 seizures per BCS and FS groups, respectively. This is particularly important given that we understand some risk factors of SUDEP, but currently available data is not sufficient to accurately predict the risk of SUDEP for individual patients. We do not know exactly how various risk factors combine and interact in potentially catastrophic ways leading to SUDEP. An important question for future research would be to understand how impaired BRS improves over time in the post-ictal state and how interactions with PGES and hypoxemia within different clusters of BCS or FS affect SUDEP. Overall, although the precise phenomenology of SUDEP is still unknown, if confirmed in future studies on SUDEP cases, post-ictal BRS measurements may provide one more quantifiable risk factor (biomarker) of SUDEP.

## Author contributions

BE and SL conceived and designed the study. BE, AD, WT, FK, KL, and SL collected the data and all authors analyzed the data. BE, SL, FK, WT, and KL wrote the paper. FK, WT, and KL developed the software.

### Conflict of interest statement

The authors declare that the research was conducted in the absence of any commercial or financial relationships that could be construed as a potential conflict of interest.
